# Outcome of Vertical Expandable Prosthetic Titanium Rib (VEPTR) Instrumentation in Scoliosis Associated With 1p36 Deletion Syndrome: A Case Report

**DOI:** 10.7759/cureus.21505

**Published:** 2022-01-23

**Authors:** Ozair Bin Majid, Mohammed A Al Rushud, Zayed Al-Zayed, Ghadeer Alsager, Jehangir A Bhat

**Affiliations:** 1 Department of Orthopaedic Surgery, King Faisal Specialist Hospital and Research Centre, Riyadh, SAU; 2 Pediatric Critical Care, Dr. Sulaiman Al-Habib Hospital, Riyadh, SAU

**Keywords:** kyphosis, syndromic scoliosis, vertical expandable prosthetic titanium rib (veptr), scoliosis, 1p36 deletion syndrome

## Abstract

Monosomy 1p36 deletion is a rare syndrome that consists of developmental delay, intellectual disability, seizures, hearing and vision defects, brain anomalies, orofacial clefting, congenital heart defects, cardiomyopathy, renal anomalies, and scoliosis. We report the case of an eight-year-old boy who presented to the orthopedic clinic with spinal deformity with a background of 1p36 deletion syndrome. The treatment modalities at this age include growing rods, vertical expandable prosthetic titanium rib (VEPTR), or posterior spinal fusion. Keeping in view the challenges in this case due to multi-organ involvement and severe intellectual disability, we decided to manage this patient with a VEPTR device to prevent the progression of scoliosis and allow spinal growth.

Vertical expandable prosthetic titanium rib (VEPTR) instrumentation for progressive scoliosis in p36 deletion syndrome is an effective mode of treatment and leads to favorable outcomes.

## Introduction

Monosomy 1p36 deletion is a chromosomal disorder characterized by severe intellectual disability and brain structural abnormalities; it is one of the most common terminals deletion syndromes in humans, with an estimated prevalence of one per 5,000 births. Most 1p36 deletion syndrome cases are isolated, and only 20% are inherited from an unaffected carrier parent. The diagnosis of this syndrome is made with fluorescence in situ hybridization (FISH) and cytogenetic analysis. The clinical picture of patients with 1p36 deletion can vary widely. Still, nearly all patients exhibit facial dysmorphic features and developmental delay with poor or absent speech, with 95% of cases having microcephaly and hypotonia. Other findings include late-closing anterior fontanel (77%), heart defects (71%), visual inattentiveness (64%), seizures (44%), skeletal abnormalities (41%), sensorineural deafness (28%), abnormal genitalia (25%) and renal abnormalities (22%) [1-3]. Generally, these patients survive until adulthood [2]. Scoliosis is relatively common in patients with 1p36 deletion syndrome, with a prevalence of 16% in one study [1,2]. In our research, we couldn't find any published papers regarding the management of scoliosis in patients with 1p36 deletion syndrome. This paper reports the successful management of progressive scoliosis in a 1p36 deletion syndrome patient using a vertical expandable prosthetic titanium rib (VEPTR) device.

## Case presentation

This is the case of an eight-year-old male patient who was first referred to our clinic four years ago with a complaint of a worsening spinal deformity. He had a cleft lip and palate that was repaired surgically when he was four months old. The patient was conceived naturally and is a full-term normal spontaneous vaginal delivery. No abnormalities or complications were reported during pregnancy. The patient suffered from multiple seizures in early childhood, but his last episode was at two years. Also, he is suffering from a global developmental delay with poor understanding and minimal speech function. He exhibited mild improvements in cognitive function over the years. The patient has been a non-walker with urinary and fecal incontinence since birth. Family history is negative for a similar presentation. Single nucleotide polymorphism array for this patient came back positive for 1p36 deletion; in addition, comparative genomic hybridization showed 1.1 duplications in the short arm of chromosome 17.

**Figure 1:**
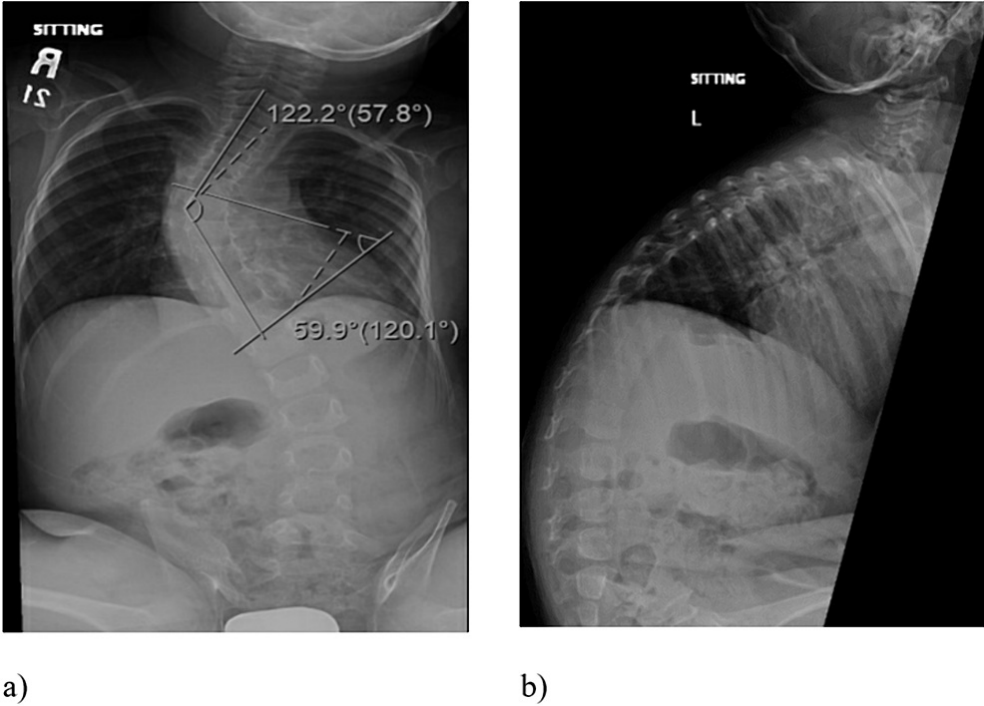
Pre-operative plain radiograph. a) A-P radiograph of the patient at three years showing scoliosis deformity. b) Lateral radiograph of the patient showing very high kyphotic angle in the thoracic spine.

This first thoracolumbar x-ray of this patient was done when he was one year old as part of a standard dysmorphic skeletal survey that showed mild dextrocurvature of the mid-thoracic spine with a curve angle measurement of 26 degrees. Over the years, his scoliosis progressed, and at the age of three, his Cobb’s angle reached 60 degrees with an increase in the kyphotic angle (Figure 1). At this point, the patient was referred to our clinic for assessment and evaluation. After a thorough assessment and considering the patient’s general medical condition, we decided to apply a vertical expandable prosthetic titanium rib (VEPTR) device. As part of the pre-op assessment, vertebral bone densitometry was performed for this patient and showed a Z-score of -4.1. MRI of the brain and spine was also performed to rule out abnormalities. No intraspinal anomalies were reported in the MRI scan.

**Figure 2:**
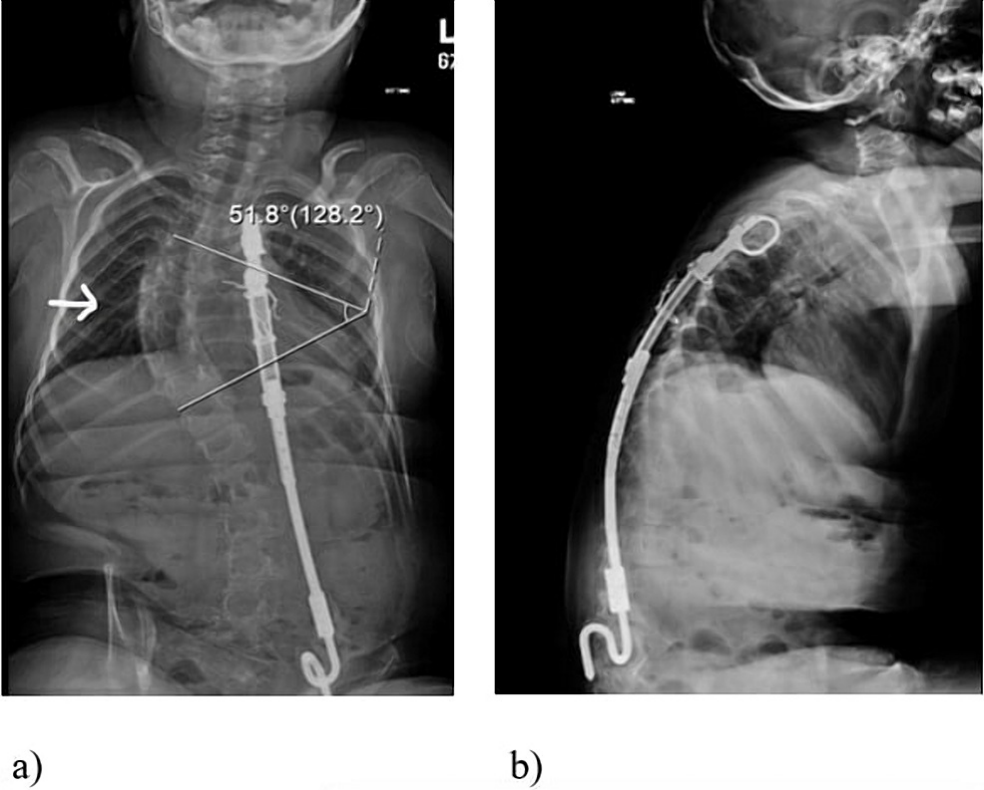
Plain radiographs at final follow-up. a) A-P radiograph of the patient at final follow-up. The arrow depicts the improvement in the patient's Cobb's angle with VEPTR in-situ. b) Lateral radiograph of the patient with VEPTR device showing improvement in thoracic kyphosis.

A rib to ilium VEPTR device was inserted with its proximal cradle in the 5^th^ and 6^th^ rib and distally resting on the iliac crest with the help of an alar hook. Two middle anchor connections through ribs were made using stainless steel wire. X-ray was done postoperatively and showed a significant improvement of Cobb's angle, measured at 40 degrees. After the initial application of VEPTR, this patient underwent four VEPTR lengthening surgeries at eight-month intervals. Over four years, the progression of scoliosis in this patient was arrested to less than 55 degrees, with the latest x-ray showing Cobb’s angle of 52. Similarly, he had a preoperative kyphotic angle of 63 degrees, which improved to 43 degrees after the index procedure and further decreased to 39 degrees after the last VEPTR lengthening procedure (Figure 2).

## Discussion

Chromosome 1p36 deletion syndrome is a group of medical signs and symptoms that manifest due to the deletion of the most distal chromosomal band on the short arm of chromosome 1 [3]. Manifestations include mental retardation and developmental delay in nearly 100% of cases, cardiac anomalies in more than 71%, and musculoskeletal in 41%. [2,4]. The most common musculoskeletal anomalies reported to be associated with 1p36 deletion syndrome are delay in the closing of the anterior fontanel (77%), hypotonia (50%), rib anomalies (16%), and scoliosis (16%) [2,4]. There is no clear prognostic information for those patients due to the clinical and genetic heterogeneity encountered, but in general, most patients seem to survive until adulthood [2,5]. Physicians must tailor their management plans for each case with appropriate diagnostic and surveillance testing [5].

Among patients with 1p36 deletion, syndrome scoliosis has been described in a few case reports with no further management details [6-10]. VEPTR implantation has been successfully used to treat early-onset scoliosis, including syndromic scoliosis such as thoracic insufficiency syndrome, Jeune syndrome, Jericho-Levin syndrome, etc. [11]. The VEPTR application aims to stop the progression of scoliosis and at the same time allow thoracic and spinal growth [12].

El-Hawary et al., in their study of 63 patients in which 4 cases were of syndromic scoliosis, successfully treated the patients with VEPTR application and noticed significant improvement at two-year follow-up. He also noticed an improvement in the thoracic kyphotic angle in these patients at a two-year follow-up [13]. Flynn et al., in a study of 24 patients treated with VEPTR device for scoliosis, noticed an 8.9-degree improvement in Cobb’s angle and 3.41 cm increase in mean thoracic height at 40 months follow up [14]. The management of syndromic scoliosis is associated with more complications than idiopathic scoliosis [15].

It is observed that complications such as neurological injury, respiratory complications, postoperative anemia, and device-related complications are more commonly seen with the treatment of syndromic scoliosis than idiopathic scoliosis. Another major complication seen with the treatment of syndromic scoliosis is pseudoarthrosis [16].

In our case, we managed the patient’s early-onset scoliosis with a VEPTR device and progressive lengthening of the device at different time intervals. We successfully corrected the Cobbs angle and stopped the curve’s progression without any major or minor complications.

## Conclusions

As 1p36 deletion syndrome is relatively common and scoliosis has been reported in these patients, and with an early presentation, we emphasize the importance of proper early investigations and follow-up. Our goal in this scenario is to arrest the progression of scoliosis with a VEPTR device and offer posterior spinal fusion once the patient has reached skeletal maturity. Furthermore, we believe that due to the multi-systemic nature of this syndrome, a multidisciplinary approach is necessary for the proper assessment and management of these patients. Given how common structural abnormalities of bone and soft tissue are in 1p36 deletion syndrome, imaging with CT and MRI before the first surgical intervention is mandatory. Finally, we recommend that further study projects should be conducted to reach a more comprehensive approach and management guidelines for scoliosis in patients with 1p36 deletion syndrome.
